# Role of Kindlin-2 in Cutaneous Squamous Carcinoma Cell Migration and Proliferation: Implications for Tumour Progression

**DOI:** 10.3390/ijms26157426

**Published:** 2025-08-01

**Authors:** Anamika Dutta, Michele Calder, Lina Dagnino

**Affiliations:** 1 Department of Physiology and Pharmacology, Western University, London, ON N6A 5C1, Canada; adutta29@uwo.ca (A.D.); michele.calder@schulich.uwo.ca (M.C.); 2 Children’s Health Research Institute, London, ON N6A 5W9, Canada; 3 Department of Oncology, Western University, London, ON N6A 5B8, Canada

**Keywords:** squamous cell carcinoma, epidermis, kindlin

## Abstract

The Kindlin family of scaffold proteins plays key roles in integrin-mediated processes. Kindlin-1 and -2, encoded by the *FERMT1* and *FERMT2* genes, respectively, are expressed in the epidermis. Kindlin-1 plays protective roles against the development of cutaneous squamous cell carcinomas (cSCCs) in epidermal keratinocytes. However, the role of Kindlin-2 in transformed epidermal keratinocytes has remained virtually unexplored. In this study, we used siRNA approaches to generate Kindlin-2-depleted cells in three isogenic transformed keratinocyte lines. PM1, MET1, and MET4 cells model, respectively, a precancerous lesion, a primary cSCC, and a metastatic lesion of the latter. MET1 cells express both Kindlin-1 and -2. However, Kindlin-1 was not detectable in PM1 and MET4 cells. *FERMT2* silencing in PM1 and MET4, but not in MET1 cells, reduced proliferation and the ability to adhere to culture surfaces and spreading. Furthermore, Kindlin-2-depleted PM1 and MET4, but not MET1 cells, exhibited decreased numbers of focal adhesions, as well as an altered F-actin and microtubule cytoskeletal organization. Significantly, *FERMT2* silencing reduced the directional migration in all three cell types. These findings are consistent with the concept that, in the absence of other Kindlin orthologues, Kindlin-2 plays a prominent role in the modulation of the proliferation, spreading, focal adhesion assembly, and motility of transformed keratinocytes, as exemplified by PM1 and MET4 cells.

## 1. Introduction

Cutaneous squamous cell carcinoma (cSCC) is the second most common human malignancy, accounting for about 20% of all skin neoplasms. Increases in the global incidence of cSCC remain unabated, largely due to chronic exposure to UV radiation and aging populations. Additional risk factors include immunosuppression, chronic inflammation, and genetic predisposition (reviewed in [1,2]). Although cSCC is readily detectable and accessible to local treatment, and only about 5% of cSCC tumours metastasize, advanced cSCC is often fatal [3,4]. cSCC also presents heterogeneous cell subpopulations within a given tumour, and the molecular mechanisms involved in its formation and progression have remained incompletely understood.

Genetic blistering diseases, such as Kindler syndrome and dystrophic epidermolysis bullosa, alter epidermal cell adhesion and interactions with the extracellular matrix and surrounding stroma, which can lead to the development of highly aggressive cSCC [5,6,7]. Kindler syndrome is caused by loss-of-function mutations in *FERMT1*, which encodes Kindlin-1 [8,9,10].

Kindlins are cytoskeleton-associated scaffold proteins that bind the cytoplasmic tail of β integrins and are indispensable for integrin activation and mechanosensing [11]. In mammals, Kindlin-1 is present in the epidermis and epithelial tissues, Kindlin-2 is broadly expressed, and Kindlin-3 is mainly restricted to the hematopoietic lineage. Despite the high homology between Kindlin-1, -2 and -3, they are functionally non-redundant [12].

The role of Kindlin proteins in tumourigenesis is complex. For example, Kindlin-1 appears to play pro-tumorigenic roles in colon and breast carcinomas, where it promotes metastasis via the integrin-dependent adhesion of circulating tumour cells to the endothelium at metastatic sites [13]. In these tumours, Kindlin-1 overexpression is associated with metastasis and poor outcomes [14,15,16,17]. In contrast, in esophageal tumours, high Kindlin-1 levels are linked to less aggressive phenotypes. In epidermal cells, Kindlin-1 can reportedly either promote or interfere with processes linked to invasiveness and motile capacity, depending on the cellular context [18,19].

Kindlin-2 is essential for embryonic development [20] and is highly expressed in many tumour types. In prostate carcinomas, it promotes testosterone-stimulated adhesion and angiogenesis [21]. Whereas in non-small cell lung carcinoma Kindlin-2 promotes epithelial-to-mesenchymal transition, it has not been detected in small cell lung tumours [22]. In breast carcinoma cells, Kindlin-2 participates in both autocrine and paracrine mechanisms that sustain tumour growth, metastasis, and pro-tumorigenic stromal–carcinoma cell interactions [23]. In these cells, Kindlin-2 additionally serves as a bridge that links β1 integrins with transforming growth factor-β (TGF-β) receptors, mediating various pro-oncogenic processes induced through the two distinct integrin- and TGF-β-mediated signaling pathways [24].

The involvement of Kindlin-1 in epidermal keratinocytes has been widely examined. In contrast, little is known about the specific roles that Kindlins fulfill in cutaneous squamous carcinomas and whether Kindlin-1 and -2 fulfill unique or overlapping functions in these tumours. To begin to address this gap, we have examined alterations in cSCC cells associated with Kindlin-2 depletion and now show that Kindlin-2 contributes to key cSCC cancer hallmark responses, including cell proliferation, spreading, and migration.

## 2. Results

### 2.1. Expression of Kindlin Proteins in Transformed Squamous Epidermal Cells

To investigate the role of Kindlin-2 in cSCC, we used isogenic patient-derived transformed epidermal cell lines that model different stages of keratinocyte transformation. Specifically, PM1 cells were generated from a precancerous actinic keratosis lesion; MET1 cells from a primary cSCC tumour; and MET4 cells from a metastatic MET1-derived lesion [25,26].

**Figure 1:**
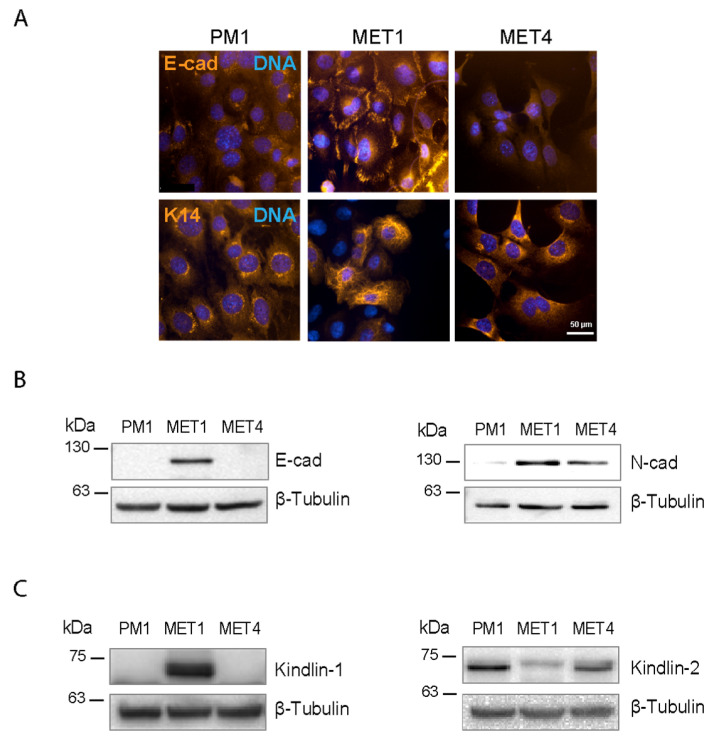
Analysis of cadherins, Keratin 14, and Kindlin proteins in transformed keratinocyte cell lines. (**A**) Representative micrographs of cells processed for immunofluorescence microscopy with antibodies against E-cadherin (E-cad) or Keratin 14 (K14). Nuclear DNA was visualized with Hoechst 33342. (**B**) Lysates from the indicated cell lines were analyzed by immunoblot with antibodies against E-cadherin (E-cad), N-cadherin (N-cad), or β-tubulin, used as loading controls. (**C**) Immunoblot analysis of cell lysates with antibodies against Kindlin-1 or Kindlin-2 and β-tubulin, used as loading control.

We first investigated the patterns of immunoreactivity of the epithelial cell markers E-cadherin and Keratin 14. In MET1 cells, E-cadherin immunoreactivity was readily observed at cell–cell junctions, and distinct Keratin 14 filaments were also detected throughout the cytoplasm (Figure 1A). In stark contrast, in PM1 and MET4 cells, E-cadherin immunoreactivity was barely detectable throughout the cytoplasm, and Keratin 14 immunoreactivity was mainly observed in a perinuclear punctate pattern (Figure 1A).

In agreement with the previous E-cadherin observations, immunoblot analysis revealed its presence in MET1 but not in PM1 or MET4 cell lysates (Figure 1B). We also assessed the presence of the EMT marker N-cadherin, readily detecting it in MET1 and MET4, but not in PM1 cell lysates (Figure 1B).

We next investigated the levels of Kindlin-1 and Kindlin-2 in these cells. Kindlin-1 is an epithelial Kindlin isoform. Significantly, although this protein was readily detected in MET1 cells, we were unable to detect it in lysates from PM1 and MET4 cells (Figure 1C). We also found that Kindlin-2 was readily detectable in all three cell lines analyzed, although MET1 cells appeared to have a somewhat lower abundance of this protein, compared to PM1 and MET4 cells (Figure 1C). These observations are consistent with a disruption of epithelial markers E-cadherin, Keratin 14, and Kindlin-1 in PM1 and MET4 but not in MET1 cells, whereas the EMT marker N-cadherin is present in MET1 and MET4 cells.

### 2.2. Effect of FERMT2 Silencing on Proliferation of PM1, MET1, and MET4 Cells

**Figure 2:**
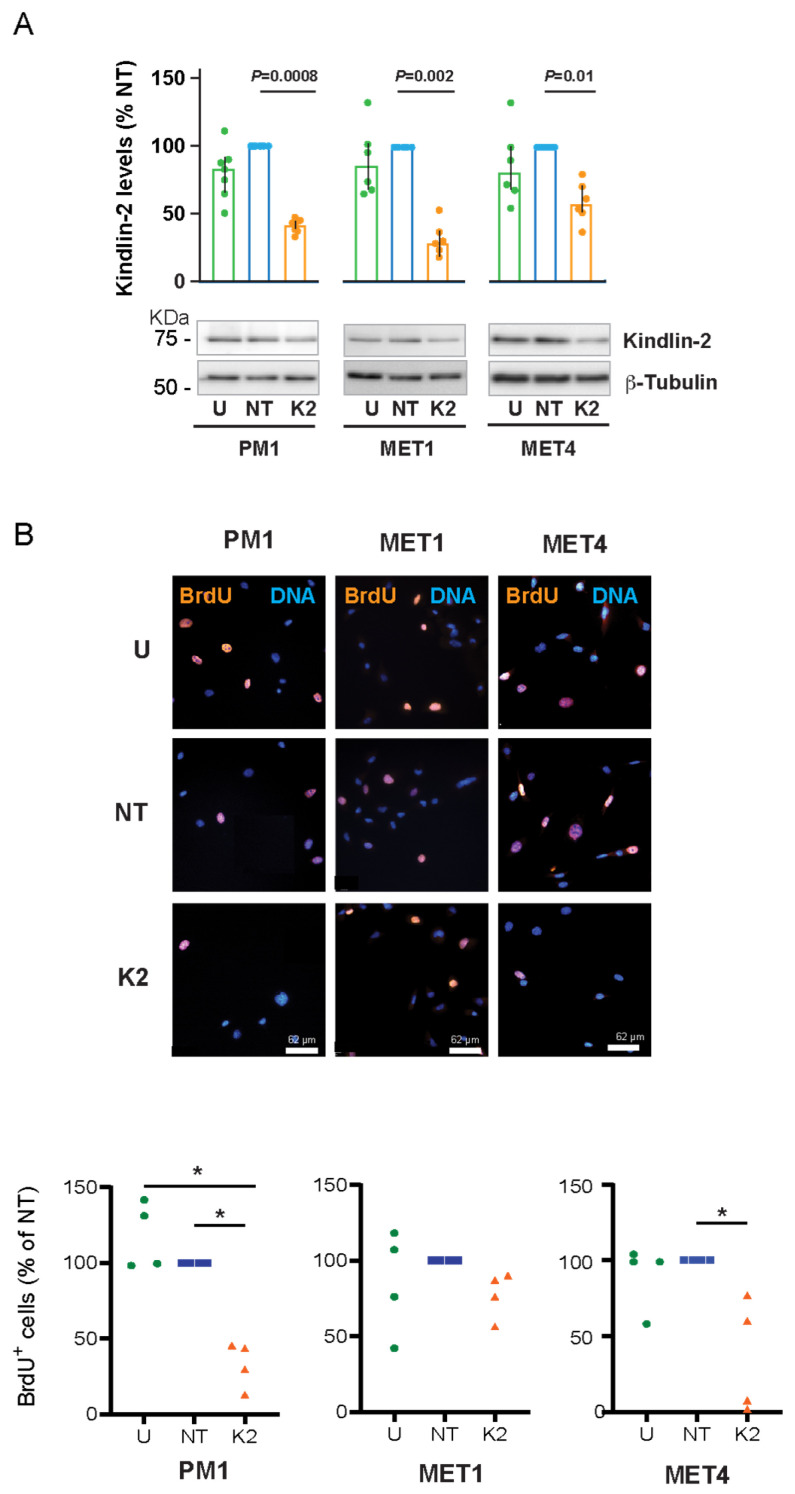
Effect of *FERMT2* silencing on cell proliferation. (**A**) The indicated cell lines were left untreated (U), transfected with either non-targeting (NT) or *FERMT2*-targeting (K2) siRNA. Protein lysates were prepared 72 h after transfection and analyzed by immunoblot with antibodies against Kindlin-2 or β-tubulin, which was used as loading control. The histograms represent Kindlin-2 levels relative to those in lysates from the corresponding NT samples, which are set to 100%. *p* values were determined with Kruskal–Wallis and post hoc Dunn’s test. Bars represent upper and lower quartile values. (**B**) The indicated cell lines were left untreated (U), transfected with either non-targeting (NT) or *FERMT2*-targeting (K2) siRNA. Sixty-six hours after transfection, the cells were trypsinized, re-seeded, and cultured for 2 h and then labelled for 6 h with BrdU, prior to processing for immunofluorescence microscopy with anti-BrdU antibodies. DNA was visualized with Hoechst 33342. The graphs represent the percentage of BrdU^+^ cells relative to those present in NT cultures, which have been set to 100%. The mean percentages of BrdU^+^ nuclei in NT PM1, MET1, and MET4 cultures were, respectively, 20.9 ± 1.6, 35.5 ± 6.9, and 17.2 ± 5.7. * *p* < 0.05 (n = 4, Kruskal–Wallis with post hoc Dunn’s test).

To assess the role of Kindlin-2 in proliferation of PM1, MET1, and MET4 cells, we silenced *FERMT2*, using siRNA pools. Analysis of Kindlin-2 protein levels either 66 h or 72 h after transfection with *FERMT2*-targeting siRNAs consistently revealed decreases of 40–90% in all three cell lines (Figure 2A, Supplemental Figure S1), without any detectable cytotoxicity.

We next analyzed 5-Bromo-2′-deoxyuridine (BrdU) incorporation into DNA. The fraction of PM1 BrdU-positive cells that were either left untreated or cultured with non-targeting siRNA was 24.6 ± 2.3% and 20.9 ± 1.6%, respectively. Significantly, only 6.9 ± 1.6% of *FERMT2*-silenced PM1 cells were BrdU-positive, which represents a 3-fold decrease relative to control cultures (Figure 2B). Similarly, whereas untreated and non-targeting siRNA-treated MET4 cultures showed 15.4 ± 1.8% and 17.2 ± 5.7% BrdU-positive cells, respectively, Kindlin-2-deficient MET4 cells exhibited a ~3-fold decrease in the fraction of BrdU-positive cells, to 6.5 ± 3.4% (Figure 2B). Finally, the fraction of BrdU-positive MET1 cells was ~30%, irrespective of whether *FERMT2* was silenced (Figure 2B). Collectively, our data show that, under the conditions of these experiments, Kindlin-2 contributes to proliferation of PM1 and MET4 cells but is not essential for MET1 cell proliferation.

### 2.3. Effect of FERMT2 Depletion on Cell Adhesion and Spreading

Kindlin proteins play key fundamental roles in promoting integrin-mediated cell adhesion to extracellular matrix substrates and spreading [27]. Thus, we next investigated the consequences of Kindlin-2 depletion on the ability of the cells to adhere. To this end, we quantified cellular lysosomal hexosaminidase activity at timed intervals following cell seeding, as it is proportional to the number of epidermal cells in a sample analyzed [28,29,30].

**Figure 3:**
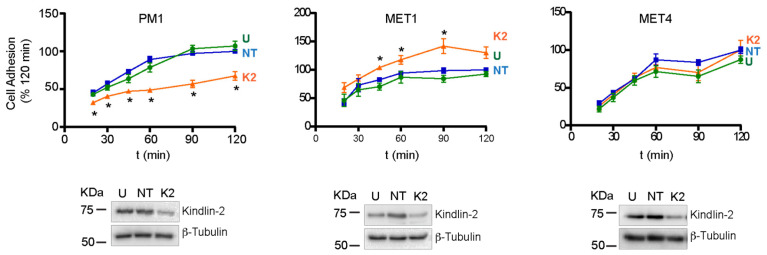
Effect of *FERMT2* silencing on cell adhesion. The indicated cell lines were left untreated (U), transfected with either non-targeting (NT) or with *FERMT2*-targeting (K2) siRNA (10 nM). Seventy-two hours after transfection, the cells were trypsinized, re-seeded, and cultured. Lysosomal hexosaminidase activity was determined at the indicated times after seeding by incubation with p-nitrophenyl-N-acetyl β-D-glucosaminide (NPAG). The data are expressed as the percentage of the absorbance values in the NPAG reaction mix relative to those obtained in cultures transfected with NT siRNA 120 min after seeding, which is set at 100%. Representative immunoblots show Kindlin-2 abundance for each treatment condition. The data are presented as mean ± SEM. * *p* < 0.05 (n = 3, two-way ANOVA with Tukey’s post hoc test).

The number of adhered untransfected PM1 cells and cells transfected with non-targeting siRNA pools displayed time-dependent increases, reaching a maximum 90 min after seeding. In contrast, Kindlin-2-deficient PM1 cells exhibited reduced adhesion, relative to control cultures (Figure 3). Significantly, no decreases in short-term adhesion were observed in either MET1 or MET4 cells, irrespective of whether they were treated with *FERMT2*-targeting siRNAs (Figure 3). Thus, under the conditions of these experiments, we found that *FERMT2* silencing results in the delayed adhesion of PM1 but not MET1 or MET4 cells.

**Figure 4:**
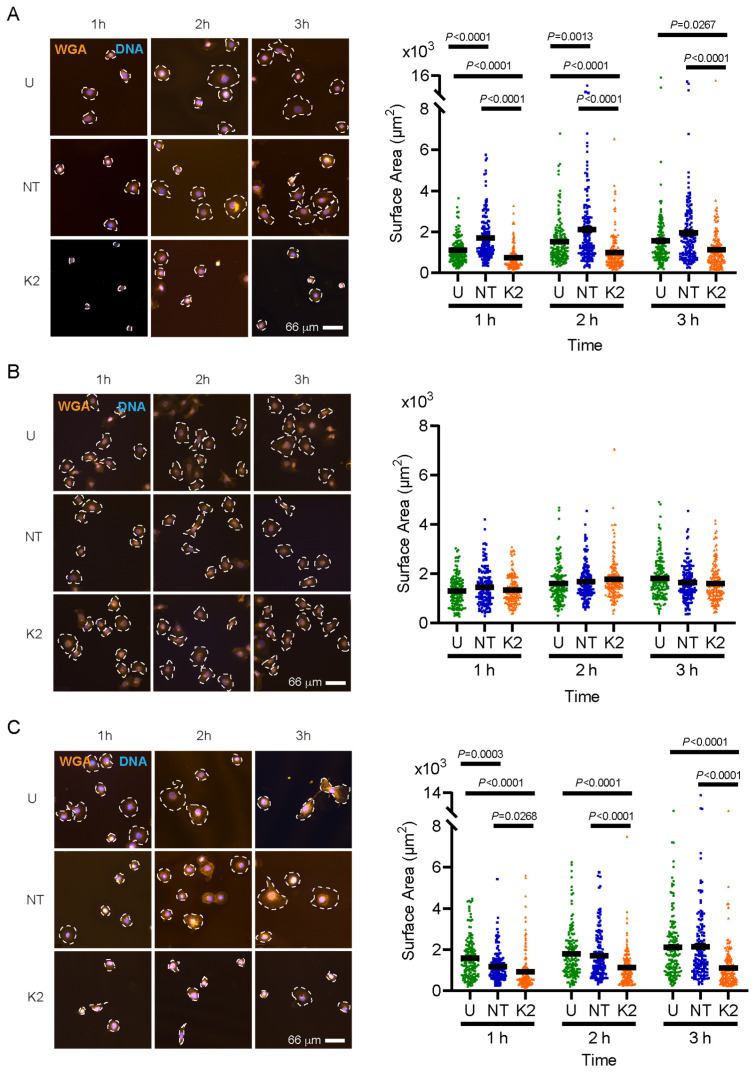
Reduction in cell surface area in Kindlin-2-depleted cells. PM1 (**A**), MET1 (**B**), and MET4 (**C**) cells were left untreated (U), transfected with either non-targeting (NT) or with *FERMT2*-targeting (K2) siRNA. Sixty-six hours after transfection, the cells were trypsinized, re-seeded, and cultured for the indicated intervals prior to processing for fluorescence microscopy using WGA and Hoechst 33342 to visualize, respectively, the plasma membrane (outlined with dashed lines) and nuclear DNA. Graphs on the right show the cell surface area measured for each cell line and treatment at the indicated time points after seeding, and are expressed as the mean ± SEM (n = 3, two-way ANOVA with Tukey’s post hoc test).

Similar experiments were conducted to evaluate the effect of Kindlin-2 depletion on the ability of the cells to spread. PM1 and MET4 cells that were untreated or transfected with non-targeting siRNA displayed increases in mean cell surface area up to 2 h after seeding. At all times analyzed during this interval, the mean surface area of Kindlin-2-deficient PM1 and MET4 cells was consistently ~50% smaller (Figure 4). In contrast, no detectable differences in spreading were observed in Kindlin-2-depleted MET1 cells relative to control cultures (Figure 4). Therefore, spreading is impaired in Kindlin-2-deficient PM1 and MET4 but not in MET1 cells.

### 2.4. FERMT2 Modulation of F-Actin Organization and Focal Adhesion Formation

**Figure 5:**
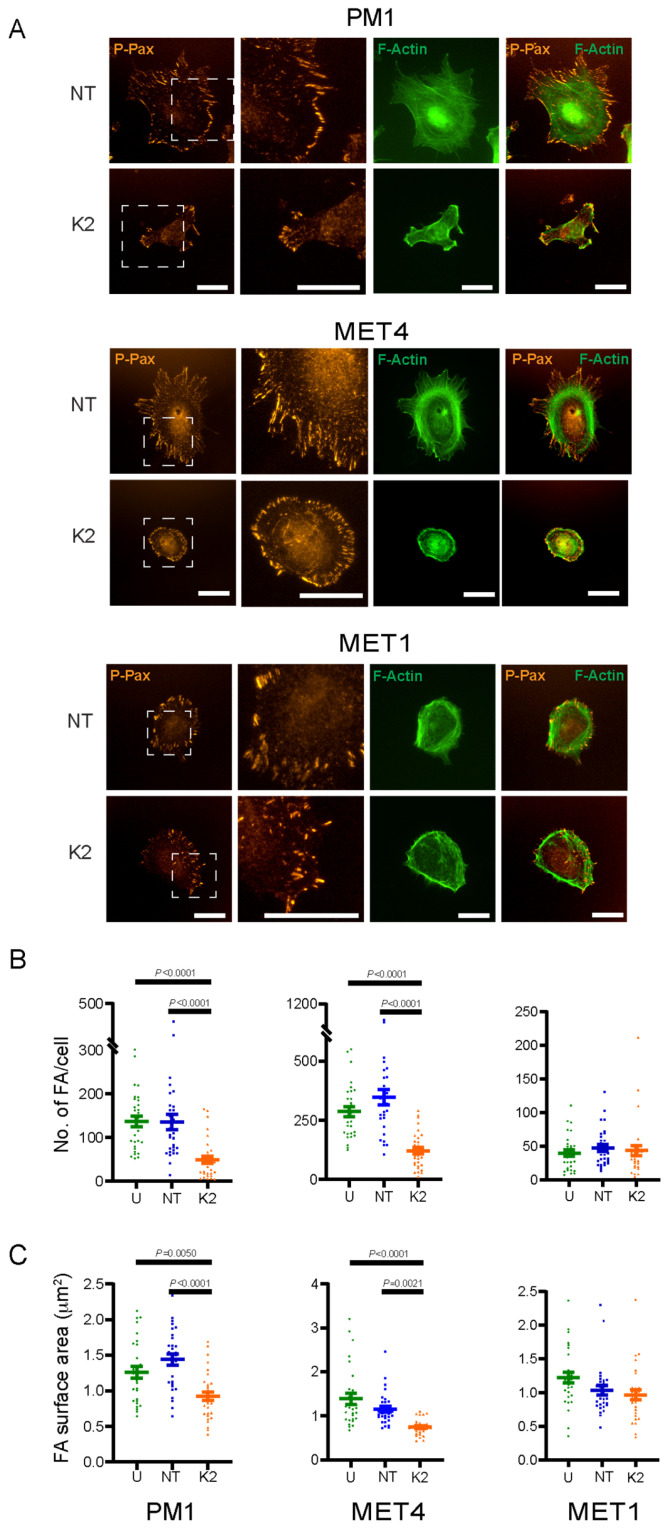
Effect of *FERMT2* silencing on the focal adhesions and F-actin organization. Cultures of the indicated cell lines were left untreated (U), transfected with either non-targeting (NT) or with *FERMT2*-targeting (K2) siRNA. Sixty-six hours after transfection, the cells were trypsinized, re-seeded, cultured for 8 h, and processed for immunofluorescence microscopy. (**A**) Phospho-paxillin (P-Pax) immunoreactivity and Alexa Fluor^®^ 488-conjugated phalloidin were used to visualize, respectively, focal adhesions and F-actin. Areas in dashed boxes are shown at higher magnification in the micrographs at right. (**B**) Number of focal adhesions/cell and (**C**) mean area of individual focal adhesions, are expressed as the mean ± SEM (one-way ANOVA, post hoc Tukey’s test, n = 3). Bar, 31 µm.

Cell adhesion and spreading are linked to actin cytoskeletal rearrangements. Given the overall impairment in spreading in *FERMT2* knockdown PM1 and MET4 cells, we next analyzed the organization of the actin cytoskeleton in these cells. Kindlin-2-expressing PM1 cells exhibited an organized F-actin network of prominent dorsal and ventral stress fibers spanning the cell body. In contrast, very few F-actin stress fibers were observed in *FERMT2* knockdown PM1 cells. Rather, thick actin bundles were detected adjacent to some membrane protrusions (Figure 5A; Supplemental Figure S2).

MET4 cells similarly exhibited a well-organized and prominent network of dorsal and ventral stress fibers spanning the cell body, as well as transverse F-actin arcs. *FERMT2* knockdown in MET4 cells resulted in a pronounced reduction in stress fibers, and those detected generally did not appear to span the cell body (Figure 5A, Supplemental Figure S3). In MET1 cells, dense cortical transverse F-actin fibers were apparent, with stress fibers that appeared to be less prominent than those observed in PM1 and MET4 cells, and no detectable alterations were observed following *FERMT2* knockdown (Figure 5A, Supplemental Figure S4).

The assembly of focal adhesions is linked to proper F-actin organization and dynamics. Hence, we next investigated if the alterations in the actin cytoskeleton observed in Kindlin-2-depleted PM1 and MET4 are accompanied by changes in focal adhesions, using phospho-paxillin immunoreactivity as a focal adhesion marker.

The mean number of focal adhesions in PM1 cells that were untreated or treated with non-targeting siRNA was, respectively, 137 ± 12.3 and 135 ± 17.4 per cell, which is significantly greater than in Kindlin-2-depleted cells, which was 48.7 ± 8.61 (Figure 5A,B; Supplemental Figure S2). The average surface area of individual focal adhesions was 1.26 ± 0.080 µm^2^ (untreated PM1 cells) and 1.44 ± 0.076 µm^2^ (non-targeting siRNA-treated PM1 cells). In contrast, a significantly reduced mean focal adhesion area (0.925 ± 0.062 µm^2^) was observed in the *FERMT2* knockdown PM1 cells (Figure 5C).

In MET4 cells, the mean numbers of focal adhesions in untreated or non-targeting siRNA cultures were similar (287 ± 21.0 and 347 ± 32.1 per cell, respectively). As observed in PM1 cells, significantly fewer focal adhesions per cell were detected in Kindlin-2-deficient MET4 cultures (122 ± 14.2; Figure 5A,B; Supplemental Figure S3). The mean surface area of focal adhesions in untreated and non-targeting siRNA-treated cells was, respectively, 1.39 ± 0.121 µm^2^ and 1.15 ± 0.068 µm^2^, whereas in the Kindlin-2-deficient cells, it was 0.744 ± 0.033 µm^2^ (Figure 5C).

Finally, no significant differences were detected among MET1 cultures that were untreated, treated with non-targeting or with *FERMT2*-targeting siRNA (Figure 5A). The mean numbers of focal adhesions per cell were, respectively, 39.2 ± 4.78, 47.2 ± 5.16, and 43.4 ± 7.59. Similarly, the average surface area of single focal adhesions in Kindlin-2-expressing control cultures was 1.21 ± 0.078 µm^2^ and 1.03 ± 0.067 µm^2^, which is not significantly different from that in Kindlin-2-deficient cells (0.965 ± 0.075 µm^2^; Figure 5B,C; Supplemental Figure S4).

### 2.5. Perturbation of Microtubule Organization by FERMT2 Silencing

**Figure 6:**
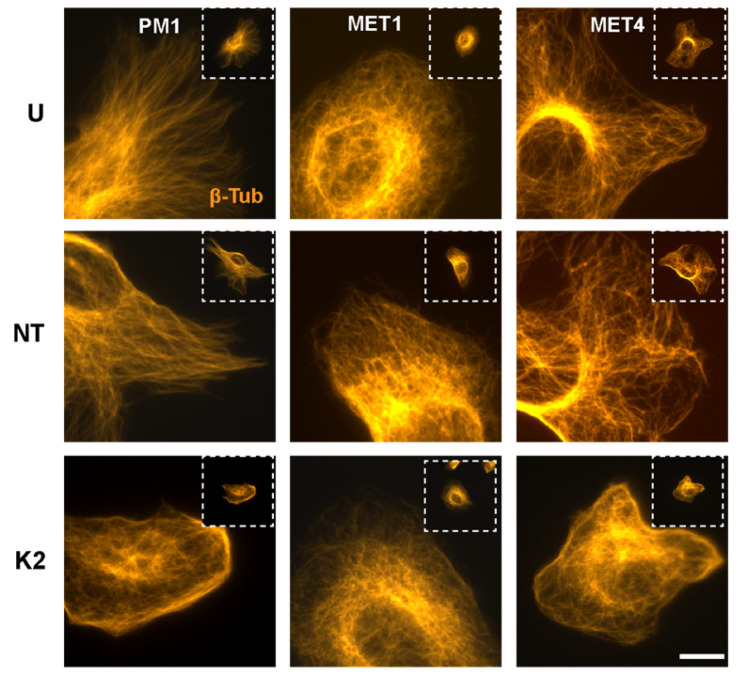
Effect of *FERMT2* silencing on the microtubule organization. Cultures of the indicated cell lines were left untreated (U), transfected with either non-targeting (NT) or with *FERMT2*-targeting (K2) siRNA. Sixty-six hours after transfection, the cells were trypsinized, re-seeded, cultured for 8 h, and processed for immunofluorescence microscopy, using anti-β-tubulin antibodies. Insets in dashed boxes correspond to lower magnification images to show the entire cell body. Bar, 16 μm.

Microtubules play important roles in focal adhesion dynamics. Reciprocally, integrins modulate the microtubule cytoskeleton, by promoting microtubule nucleation, growth, and stabilization [31]. We found that microtubule networks in Kindlin-2-expressing PM1 and MET4 cells appeared as long filaments that extended radially from perinuclear regions toward the cell periphery (Figure 6; Supplemental Figure S5). In contrast, microtubules in *FERMT2* knockdown PM1 and MET4 cells were perturbed and characterized by the presence of thick cortical microtubule filaments parallel to the plasma membrane or visualized as a reticular network denser around the nucleus, but also visible throughout the cytoplasm (Figure 6; Supplemental Figure S5). MET1 cells showed a microtubule network that reached areas adjacent to the plasma membrane, with very few, if any, cortical filaments, irrespective of whether they had been treated with *FERMT2*-targeting siRNA (Figure 6; Supplemental Figure S5). Together, our observations indicate that Kindlin-2 depletion alters the steady-state abundance and size of focal adhesions, as well as the F-actin and the microtubule cytoskeletons.

### 2.6. Kindlin-2 Depletion Decreases Forward Cell Migration

The observed alterations in focal adhesions and cytoskeletal filaments, as well as impaired cell spreading, prompted us to explore the consequences of *FERMT2* silencing on forward cell migration. To this end, confluent PM1, MET1, and MET4 monolayers were scrape-wounded, and cell movements into the cell-free area over time were recorded by time-lapse videomicroscopy. The changes in the wound area at timed intervals after scraping were then measured.

**Figure 7:**
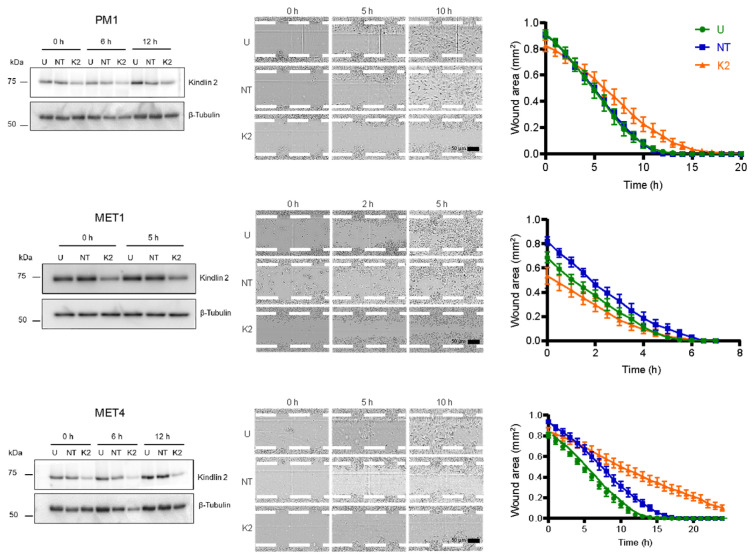
*FERMT2* silencing reduces forward cell migration. The indicated cell lines were left untreated (U), transfected with either non-targeting (NT) or with *FERMT2*-targeting (K2) siRNA (10 nM). Sixty-six hours after transfection, the cells were trypsinized, re-seeded for migration measurements and cell lysate preparation, and cultured for 2 h. Confluent monolayers were used for cell lysate preparation or were scrape-wounded, and time-lapse videomicroscopy images were then acquired. The data represent the fraction of cell-free surface area values at the indicated times after wounding (mean ± SEM, n = 3). Bars, 50 μm.

PM1 cells untreated or transfected with non-targeting siRNA migrated to close the cell-free area by 12 h after wounding, at an approximate rate of 0.059 mm^2^/h. In contrast, PM1 cells treated with *FERMT2*-targeting siRNA closed the wound area with a considerable delay at 18 h (~0.083 mm^2^/h; Figure 7).

Similarly, MET4 cells that had been left untreated or were transfected with non-targeting siRNA pools migrated to completely cover the wound area by 15 h and 18 h, respectively, a difference that was not statistically significant. In contrast, MET4 cells treated with *FERMT2*-specific siRNA pools had 30% and 12% of the cell-free area remaining 18 h and 24 h after scraping, respectively (Figure 7). The rate at which Kindlin-2-deficient MET4 cells covered the wound area was 0.034 mm^2^/h, which was about 50% lower compared to both Kindlin-2-expressing controls (0.064 and 0.062 mm^2^/h). Finally, MET1 cells that were untreated or that were transfected with non-targeting siRNA migrated to cover the wound area at estimated rates of 0.158 mm^2^/h and 0.143 mm^2^/h, respectively. Kindlin-2-deficient MET1 cells exhibited a small but significant reduction in forward migration, as their rate of wound closure was 0.103 mm^2^/h (Figure 7). These results demonstrate that Kindlin-2 is important to support PM1, MET1, and MET4 forward cell migration.

## 3. Discussion

In this study, we have explored functional roles that Kindlin-2 fulfills in transformed epidermal keratinocytes, using cell lines that model cSCC progression stages, from pre-cancerous actinic keratosis (PM1 cells) to primary cSCC (MET1 cells) and to metastatic cSCC (MET4 cells). Although the functions of Kindlin-2 have been documented in several non-epithelial tissues, they have remained virtually unexplored in transformed epidermal cells.

Using siRNA-induced downregulation of Kindlin-2 in transformed epidermal cells, we demonstrate that Kindlin-2 contributes to focal adhesion and F-actin cytoskeletal modulation, with functional consequences on cell proliferation and migration.

Normal epidermal keratinocytes express Kindlin-1, which is restricted to epithelial tissues, as well as Kindlin-2. Significantly, we readily detected Kindlin-1 in MET1 cells but not in PM1 or in MET4 cells. Examination of the cBioPortal database indicates a negligible incidence of *FERMT1* somatic mutations (<0.1%) in the reported cSCC samples analyzed. Kindlin-1 is also reportedly highly expressed in actinic keratoses and primary cSCC specimens [18]. Whether the changes in the *FERMT1* expression observed in PM1 and MET4 cells arose in the original lesions or developed during adaptation to cell culture remains unclear, and establishing whether the observed changes arise from transcriptional or post-transcriptional alterations will be an important area for future research. Regardless, PM1 and MET4 cells constitute excellent models to specifically examine the biological roles that Kindlin-2 plays in transformed epidermal keratinocytes, without potentially overlapping contributions from Kindlin-1. It is noteworthy that the absence of Kindlin-1 observed in PM1 and MET4 cells is also accompanied by alterations in E-cadherin and Keratin 14. Both cSCC lines express N-cadherin, but the transition from MET1 to MET4 cells appears to include the loss of E-cadherin, potentially suggesting that the loss of Kindlin-1 may be associated with the development of a more pronounced epithelial-to-mesenchymal phenotype in transformed epidermal cells.

We found that Kindlin-2 is essential for efficient spreading in a Kindlin-1-deficient PM1 and MET4 cell background, highlighting the critical role that Kindlins play in this process. Similar observations have been reported in cultured primary mouse keratinocytes and in immortalized human keratinocyte lines [32,33]. Significantly, Kindlin-2 knockdown in MET1 cells did not appreciably affect spreading, suggesting overlapping roles of Kindlins 1 and 2 in this aspect of cSCC biology. Defects in cell spreading in Kindlin-2-depleted cells have been reported in primary dermal fibroblasts, immortalized human keratinocytes, as well as in prostate and in breast carcinoma cells [21,33,34,35], highlighting potential differences in Kindlin-2 functions that may depend not only on the tissue type but also on the transformation status of cells within a given tissue.

Cell morphology and spreading over extracellular matrix substrates are modulated by dynamic changes in the F-actin cytoskeleton and in the microtubule network. Kindlin-2 binds F-actin directly and indirectly, modulating bidirectional integrin signaling in various cell types [20,36]. The reduction in visible F-actin stress fibers we observed upon Kindlin-2 depletion in PM1 and MET4 cells is reminiscent of similar changes reported in other cell types. The F-actin aggregates observed in some areas adjacent to the plasma membrane in PM1 and MET4 cells also appear similar to the F-actin alterations reported in Kindlin-2-depleted C2C12 myofibroblasts and podocytes, although they differ from the reported pronounced decrease in peripheral F-actin described in primary dermal fibroblasts [35,36,37]. Significantly, the organization of microtubules was also markedly perturbed in Kindlin-2-depleted PM1 and MET4. In normal epidermal keratinocytes, Kindlin-1 contributes to microtubule stability [38]. Our observations now show that Kindlin-2 also contributes to the maintenance of normal microtubule networks in transformed epidermal cells. Microtubule stability in primary epidermal keratinocytes is modulated, in part, through the activation of Rac1 [39], and, significantly, Kindlin-2 has been shown to contribute to Rac1 activation in mesenchymal cells. An important area for future research will be to determine whether the microtubule abnormalities observed in Kindlin-2-depleted PM1 and MET4 cells are associated with alterations in microtubule stability and Rho GTPase activation.

Kindlin-2 associates with β1 integrins to promote focal adhesion assembly in primary mouse epidermal keratinocytes and other cell types [32,40]. Kindlin-2 concentrates at focal adhesion regions proximal to the plasma membrane by virtue of its capacity to bind directly to β1 integrins, as well as to other proteins present in mature focal adhesions, such as paxillin [40]. Kindlin-2 contributions to focal adhesion formation are also required for efficient cell spreading [32]. Kindlin-2 depletion in PM1 and MET4 cells resulted in pronounced decreases in the focal adhesion numbers and size, with no detectable alterations in MET1 cells. This is consistent with the concept of potentially overlapping functions of Kindlin-1 and Kindlin-2 in focal adhesion assembly in transformed keratinocyte cell lines. An important area for future research will be to investigate whether direct interactions between Kindlin-2 and β-integrins are essential for these processes in the cSCC cell models we have characterized.

Kindlin-2 downregulation in PM1 and MET4 cells is also associated with decreased forward motility. These decreases mirror those reported in non-transformed keratinocytes and other cell types, but contrast with reported observations in HT1080 fibrosarcoma cells, in which the loss of Kindlin-2 promotes rear focal adhesion disassembly and increased migration [27]. It will be important to further characterize the relative roles that Kindlins 1 and 2 fulfill in focal adhesion dynamics in epidermal keratinocytes.

cSCC is a human tumour with one of the highest mutational burdens [41]. For this reason, metastatic cSCC remains a major therapeutic challenge. The pathogenesis mechanisms involved in progression from actinic keratosis to the various stages of cSCC are regulated by a combination of complex genetic, epigenetic, environmental, and molecular processes. Oncogenic drivers in cSCC include those that alter cell cycle progression, apoptosis, and EMT [42]. Our studies have identified Kindlin-2 as a positive modulator of both proliferation and migration in transformed keratinocytes and suggest that those contribution may be amplified in Kindlin-1-deficient cell backgrounds. Thus, Kindlin-family-modulated processes may be useful targets in combination therapies directed at multiple dysfunctional pathways in a cSCC tumour.

In summary, our results show that the Kindlin-2 downregulation in PM1 and MET4 cells is associated with alterations in hallmark features of cancer, including decreased cell proliferation and migration. Elevated Kindlin-2 levels in keratinocyte and other epithelial carcinomas, such as esophageal SCC, are associated with a poor prognosis and reduced overall survival [43]. Thus, Kindlin-2 may function as a phenotype modifier in cSCC, imparting selective advantages for cSCC progression and constituting a potential target for anticancer therapies in the epidermis.

## 4. Materials and Methods

### 4.1. Cell Culture

PM1 cells were derived from a precancerous dysplastic lesion on the patient’s forehead, MET1 cells were derived from a primary invasive cSCC growing on the patient’s right hand, and MET4 cells were established from an axillary lymph node metastasis that developed from the primary MET1 tumour [44]. These cells were purchased from Ximbio (PM1 #153571, MET1 #153539, MET4 #153570) and last re-authenticated in Fall 2023. The cells were cultured in Dulbecco’s Modified Eagle Medium (DMEM; Wisent Bioproducts, Saint-Jean-Baptiste, QC, Canada, 319-005-CL) supplemented with 8% (*v*/*v*) fetal bovine serum (FBS; Life Technologies, Carlsbad, CA, USA, 12483-020) and confirmed to be mycoplasma-free (quarterly test intervals) using MycoAlert™ Plus Mycoplasma Detection Kits (Lonza, Basel, Switzerland, L707-7610). All experiments were conducted with cells that had been passaged for less than 20 generations.

### 4.2. Antibodies

Antibodies, their sources, and application details are listed in Table 1.

### 4.3. siRNA Transfection

siRNA transfections were conducted as described [30]. Briefly, cells were seeded onto 6-well culture plates, at a density of 3.5 × 10^4^ PM1, 1.0 × 10^5^ MET1, or 4.5 × 10^4^ MET4 cells/well. Twenty-four hours after plating, the cells were transfected with siRNA pools (ON-TARGETplus siRNA SMARTpool Dharmacon, Horizon Discovery, Lafayette, CO, USA), targeting *FERMT2* (10979 siRNA SMARTpool, L-012753-00-0020) or non-targeting siRNA pools (ON-TARGETplus Non-targeting Pool, Dharmacon, D-001810-10-05). The final siRNA concentration was 30 nM, unless otherwise indicated in individual experiments, and the transfection reagent used was Dharmafect 1 (Dharmacon, T-2001-02). Transfected cells were cultured at 37 °C for 66 or 72 h, as indicated in individual experiments.

### 4.4. Cell Lysates and Immunoblot Analysis

Lysates from PM1, MET1, and MET4 cells were prepared with a modified RIPA buffer, as described [45,46,47]. Cell monolayers in each well of a 6-well culture plate were lysed with 200 µL of ice-cold RIPA buffer (50 mM Tris pH 7.6, 150 mM NaCl, 1% Triton X-100) supplemented with freshly added phenylmethanesulfonyl fluoride (PMSF; 1 mM, Bioshop, Burlington, ON, Canada, PMS123), Na_3_VO_4_ (5 mM Bioshop, SOV664), NaF (5 mM, Millipore Sigma, St. Louis, MO, USA, S7920), aprotinin (1 mg/mL, Bioshop, APR.600.10), leupeptin (1 mg/mL, Bioshop, LEU001), and pepstatin (1 mg/mL Bioshop, PEP605). Protein concentration in the lysates was determined using Bradford assays (Bio-Rad Protein Assay Dye Reagent Concentrate, Bio-Rad Laboratories, Hercules, CA, USA, 500-0006), following the manufacturer’s instructions. Proteins in lysates were resolved by denaturing gel electrophoresis and transferred to 0.2-μm polyvinylidene fluoride membranes, which were probed with antibodies indicated in individual experiments. For every experiment reported herein, the decrease in cellular Kindlin-2 levels in *FERMT2*-targeting siRNA-treated cultures was verified to be ≥50%, using immunoblot analysis.

### 4.5. Measurements of Cell Proliferation

Incorporation of 5-Bromo-2′-deoxyuridine (Fisher Scientific, Fairlawn, NJ, USA, H27260.03) into DNA was conducted as described [48], with minor modifications. Cells that had been cultured in the presence or absence of siRNA pools for 66 h were trypsinized and resuspended in growth medium at a density of 16.7 × 10^3^ cells/mL, and 300 μL (5000 cells/well) of this cell suspension was added to replicate wells of an 8-well μ-Slide (ibidi, Gräfelfing, Germany, 8006). The cells were cultured for 2 h at 37 °C to allow attachment, at which time the medium was replaced with 300 µL of pre-warmed (37 °C) medium containing 20 µM BrdU. The cells were then incubated for 6 h at 37 °C, fixed in freshly diluted 4% paraformaldehyde (PFA, 40 min, 22° C), and permeabilized in 0.1% Triton-X 100 diluted in phosphate-buffered saline (PBS). Nuclear DNA was denatured by incubation in 2 M HCl (20 min, 22° C). The cells were probed with anti-BrdU antibodies, and the fraction of cells exhibiting BrdU immunoreactivity was determined from immunofluorescence micrographs as described [49].

### 4.6. Immunofluorescence Microscopy

Sixty-six hours after siRNA transfection, cells were trypsinized, resuspended in growth medium, and seeded at a density of 5000 cells/well onto 8-well μ-Slides and cultured at 37 °C for 1–6 h prior to fixation with freshly diluted 4% (*w*/*v*) PFA for 20 min at 22 °C, followed by permeabilization with 0.1% (*v*/*v*) Triton X-100 and 0.1% (*w*/*v*) bovine serum albumin (BSA) in PBS with rocking. The cells were rinsed twice with 300 µL of PBS, incubated with blocking solution (PBS with 5% (*w*/*v*) BSA) for 1 h at 22 °C, and incubated overnight at 4 °C with primary antibody. After three PBS washes, samples were probed with AlexaFluor™-conjugated secondary antibody at 22 °C for 1 h, washed thrice with PBS, incubated with Hoechst 33342 (20 μg/mL; Thermo Fisher Scientific, Waltham, MA, USA, H1399) for 15 min at 22 °C, rinsed thrice with PBS, and mounted with Immu-Mount^TM^ mounting medium (Thermo Fisher Scientific, 77-86-1). Fluorescence images were obtained with a Leica DMIRBE fluorescence microscope (Leica, Wetzlar, Germany) equipped with an ORCA-ER digital camera (Hamamatsu Photonics, Shizuoka, Japan), using Volocity 6.1.1 software (Improvision, Coventry, England). For experiments with wheat germ agglutinin (WGA), cells were fixed with 4% PFA and immediately incubated with Alexa Fluor™ 555-conjugated WGA (5.0 μg/mL; Thermo Fisher Scientific, W32464) for 10 min at 22 °C. For experiments to visualize the actin cytoskeleton, cells were fixed, permeabilized, and incubated with Alexa Fluor™ 488-conjugated phalloidin in PBS (1:100, *v*/*v*) at 22 °C for 30 min prior to incubation with Hoechst 33342.

### 4.7. Measurement of Cell Adhesion

Cell adhesion was measured as described [30]. Cultures transfected with siRNA pools for 72 h were trypsinized, resuspended in growth medium at a density of 3 × 10^5^ cells/ml, and seeded in triplicate onto 96-well cell plates (3 × 10^4^ cells/well). At timed intervals after plating, unattached cells were removed by aspiration, and the attached cells were incubated in 50 mM sodium citrate buffer, pH 5.0, containing 0.025% Triton X-100 and 3.75 mM p-nitrophenyl-N-acetyl β-D-glucosaminide (NPAG; Millipore Sigma, N9376) for 4 h at 37 °C. Lysosomal hexosaminidase activity was stopped by addition of 50 mM glycine buffer, pH 10.4, containing 5 mM EDTA (75 μL/well). Absorbance at 405 nm was measured with a Tecan Spark^®^ Multimode Microplate reader (Tecan Austria GmbH, Grödig, Austria). Absorbance values between 0 and 2 were read, ensuring that the assay was conducted within a linear range of values.

### 4.8. Analysis of Cell Surface Area and Focal Adhesions

Cell surface area was measured from photomicrographs of WGA-labelled cells, using ImageJ version 2.14. To this end, scales were calibrated, the parameter to be measured was set as “Area”, and brightness and contrast of the micrographs were adjusted to allow clear visualization of the plasma membrane, as defined by WGA fluorescence. The plasma membrane of individual cells was outlined with the Freehand tool. Area measurements (expressed in μm^2^) of 50 cells in each treatment group, for each biological replicate, were obtained for further analyses.

The analysis of focal adhesions was conducted from micrographs showing phosphorylated paxillin immunoreactivity, using ImageJ version 2.14, as described [50,51].

### 4.9. Analysis of Cell Migration

Sixty-six hours after siRNA transfection, cells were trypsinized and resuspended in growth medium at a density of 4 × 10^5^ cells/mL, and 4 × 10^4^ cells/well were seeded in quadruplicate samples onto Incucyte^®^ 96-well cell culture plates (Fisher Scientific, 361057313). The cells were allowed to adhere to the culture surface for 2 h at 37 °C. Confluent monolayers were then scraped using an Incucyte^®^ 96-Well Woundmaker Tool (Sartorius, Göttinge, Germany, BA-04858). Migration into the denuded area of cells cultured at 37 °C was monitored by acquiring phase-contrast time-lapse images following wounding, using an Incucyte S3 imager (Sartorius), with the Incucyte Scratch Wound Analysis Software Module Version 2020-A (Sartorius). Image sequences were imported into ImageJ software (version 2.14) [52], using the MRI Wound Healing plugin to measure changes in cell-free wound area over time.

### 4.10. Statistical Analyses

Statistical analyses were conducted with GraphPad Prism (version 10.4.2, GraphPad Software Inc., La Jolla, CA, USA). Statistical tests to determine *p* values and sample size are specified in the legends to figures. For all experiments, significance was set at *p* < 0.05.

## Figures and Tables

**Table 1:** Antibodies used in these studies.

Antibody	Dilution	Source
β-Tubulin	IB 1:3000 IF 1:100 ^1^	Developmental Studies Hybridoma Bank, University of Iowa, Iowa City, IA, USA (E7)
BrdU	IF 1:100	Developmental Studies Hybridoma Bank (G3G4)
Cytokeratin 14	IF 1:250	Novus Biologicals, Centennial, CO, USA (LL002)
E-cadherin	IF 1:250	Cell Signaling Technology Danvers, MA, USA (14472)
E-cadherin	IB 1:1000	BD Biosciences, San Jose, CA, USA (610182)
Kindlin-1	IB 1:1000	Cell Signaling Technology (36734S)
Kindlin-2	IB 1:1000	EMD Millipore, St. Louis, MO, USA (MAB2617)
N-cadherin	IB 1:1000	BD Biosciences (610921)
Phospho-paxillin (Tyr118)	IF 1:100	Cell Signaling Technology (69363S)
HRP-conjugated anti-mouse IgG	IB 1:5000	Jackson ImmunoResearch, West Grove, PA, USA (115-135-003)
HRP-conjugated anti-rabbit IgG	IB 1:5000	Jackson ImmunoResearch (111-035-144)
AlexaFluor^TM^ 488-conjugated anti-mouse IgG	IF 1:250	Thermo Fisher Scientific, Waltham, MA, USA (A-11001)
AlexaFluor^TM^ 594-conjugated anti-mouse IgG	IF 1:250	Jackson ImmunoResearch (115-585-062)
AlexaFluor^TM^ 555-conjugated anti-rabbit IgG	IF 1:250	Thermo Fisher Scientific (A-21428)

^1^ IB, immunoblot; IF, immunofluorescence microscopy.

## Data Availability

All data supporting the reported results are included in the manuscript.

## Supplementary Materials

The following supporting information can be downloaded at https://www.mdpi.com/article/10.3390/ijms26157426/s1.

## Abbreviations

The following abbreviations are used in this manuscript:

BrdU	5-Bromo-2′-deoxyuridine
BSA	Bovine serum albumin
cSCC	Cutaneous squamous cell carcinoma
DMEM	Dulbecco’s modified minimum essential medium
EDTA	Ethylenediaminetetracetic acid
FBS	Fetal bovine serum
NPAG	p-Nitrophenyl-N-acetyl β-D-glucosaminide
PBS	Phosphate-buffered saline
PFA	Paraformaldehyde
PMSF	Phenylmethanesulfonyl fluoride
TGF-β	Transforming growth factor-β
WGA	Wheat germ agglutinin
