# Supplementary material for: Role of Kindlin-2 in Cutaneous Squamous Carcinoma Cell Migration and Proliferation: Implications for Tumour Progression
Source: Int J Mol Sci. 2025 Aug 1;26(15):7426. doi: 10.3390/ijms26157426 (PMC12347749; doi:10.3390/ijms26157426)
Supplement: Supplementary file 1 [file ijms-26-07426-s001.zip › ijms-3746234-supplementary.pdf]

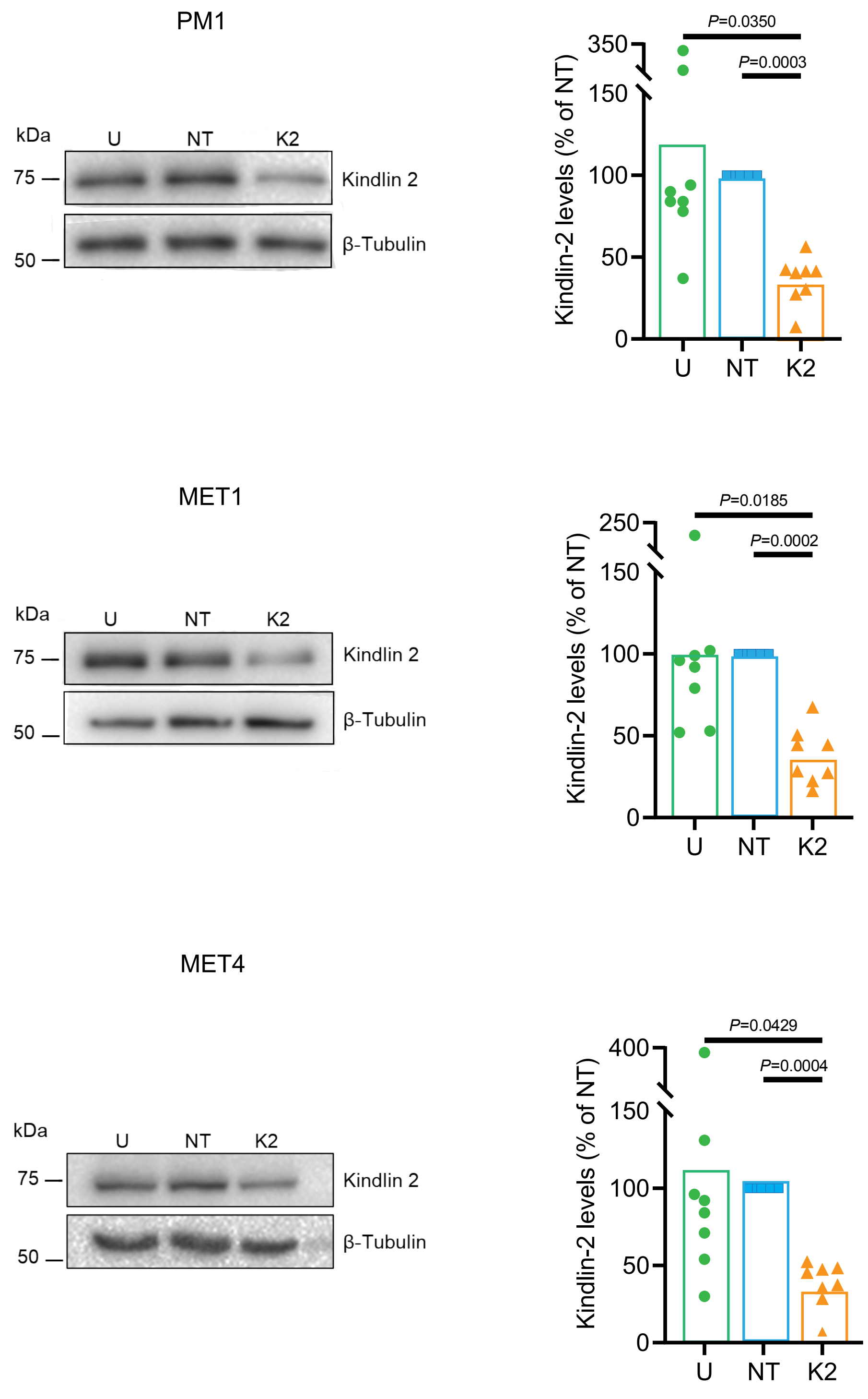

Supplemental Fig. S1. Effect of *FERMT2* silencing on Kindlin-2 abundance. The indicated cell lines were left untreated (U), transfected with either non-targeting (NT) or *FERMT2*-targeting (K2) siRNA. Protein lysates were prepared 66 h after transfection and analyzed by immunoblot with antibodies against Kindlin-2 or β-tubulin, which was used as loading control. The histograms at right represent Kindlin-2 levels relative to those in lysates from the corresponding NT samples,which are set to 100%. P values were determined with Kruskal-Wallis test and post-hoc Dunn’s test.

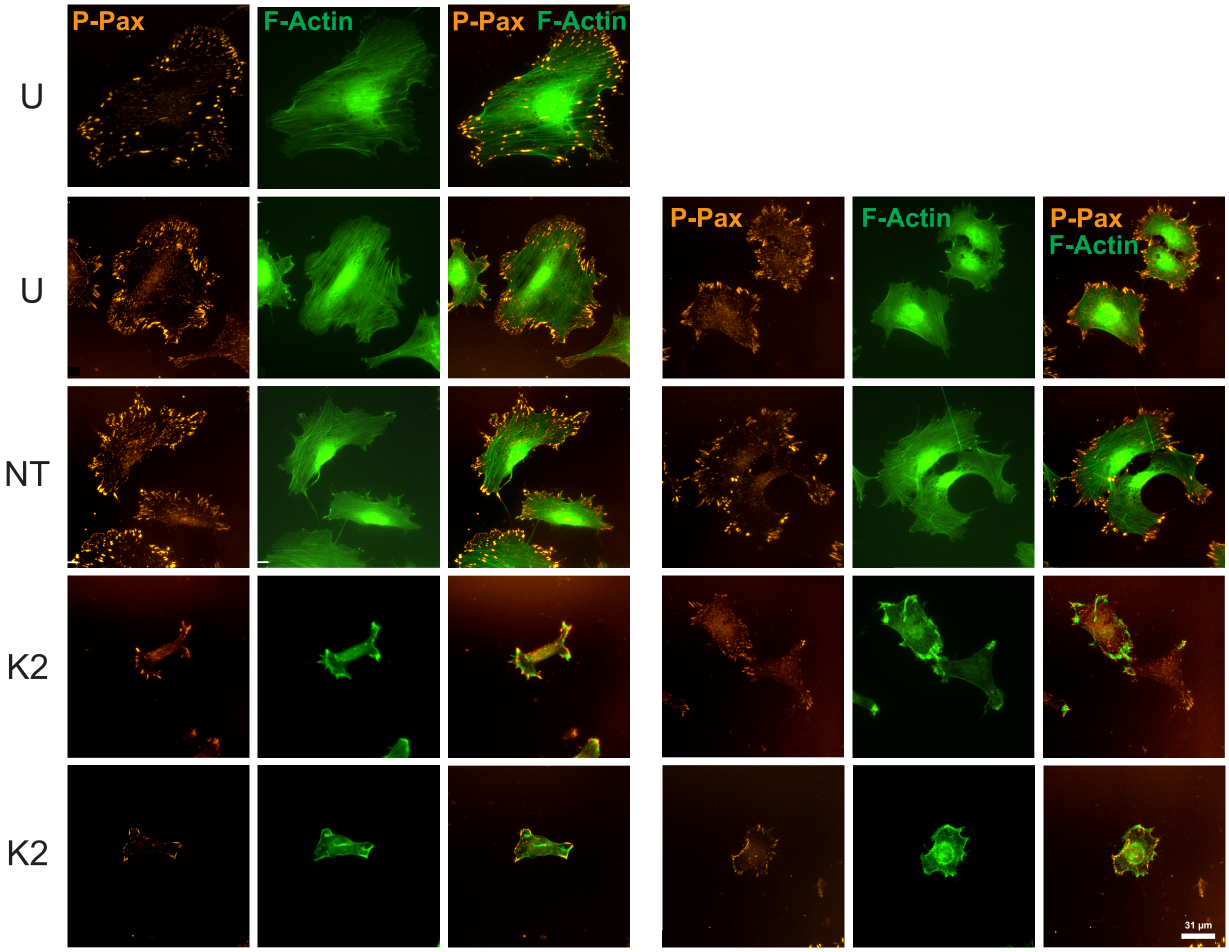

Supplemental Fig. S2. Effect of *FERMT2* silencing on focal adhesions and F-actin organization. PM1 cultures were left untreated (U), transfected with either non-targeting (NT) or with *FERMT2*-targeting siRNA. Sixty-six hours after transfection, the cells were trypsinized, re-seeded, cultured for 8 h and processed for immunofluorescence microscopy. Phospho-paxillin (P-Pax) immunoreactivity and Alexa Fluor 488®-conjugated phalloidin were used to visualize, respectively, focal adhesions and F-actin.

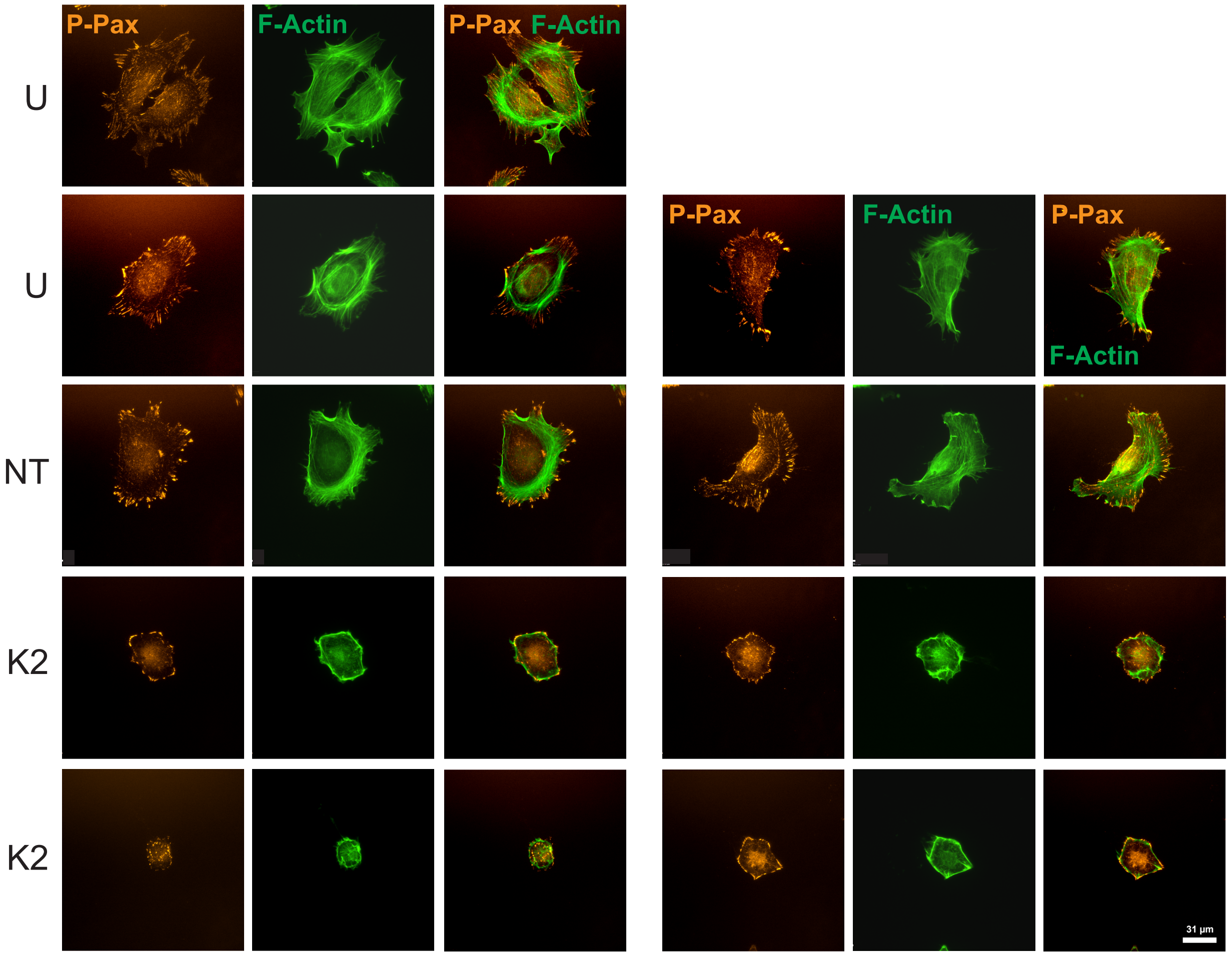

Supplemental Fig. S3. Effect of *FERMT2* silencing on focal adhesions and F-actin organization. MET4 cultures were left untreated (U), transfected with either non-targeting (NT) or with *FERMT2*-targeting siRNA. Sixty-six hours after transfection, the cells were trypsinized, re-seeded, cultured for 8 h and processed for immunofluorescence microscopy. Phospho-paxillin (P-Pax) immunoreactivity and Alexa Fluor 488®-conjugated phalloidin were used to visualize, respectively, focal adhesions and F-actin.

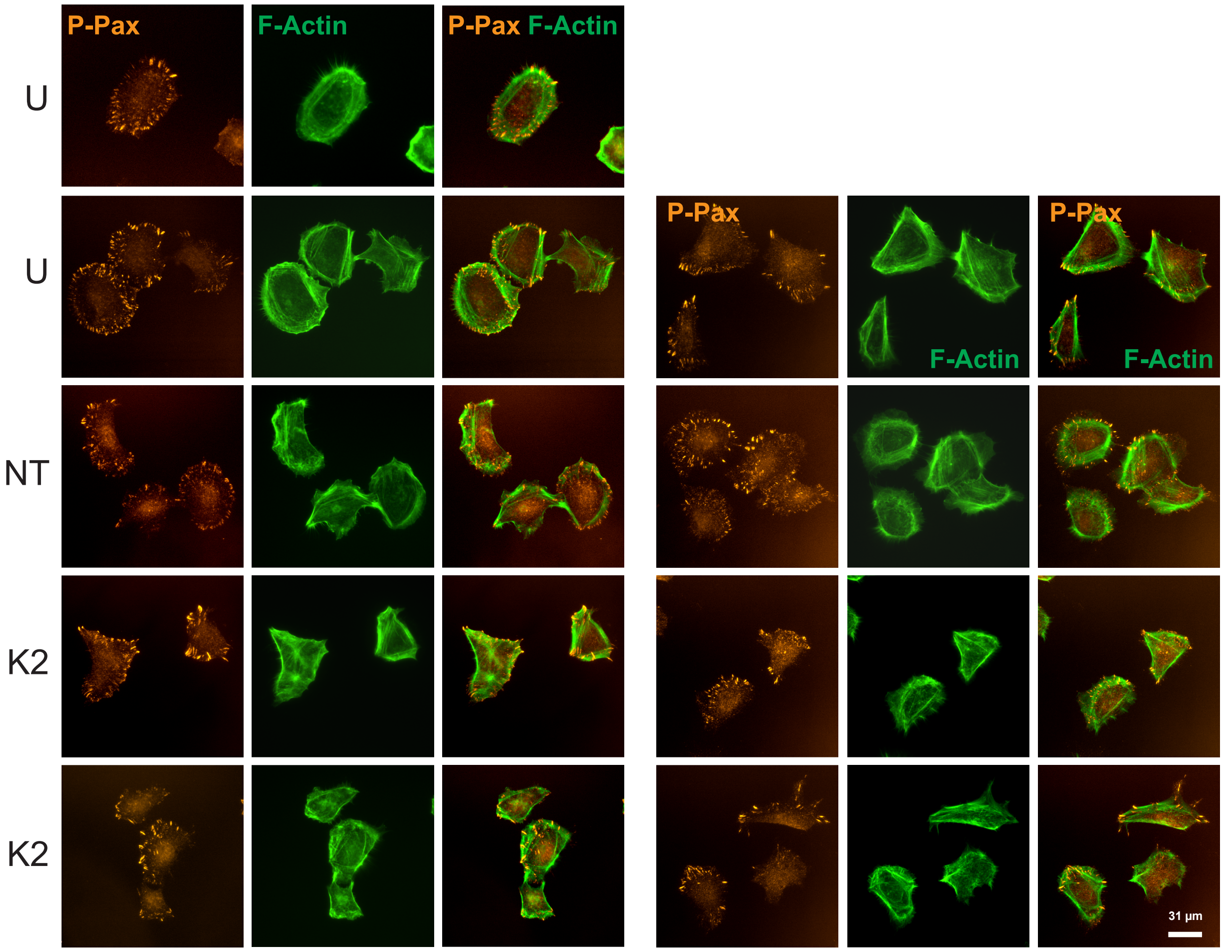

Supplemental Fig. S4. Effect of *FERMT2* silencing on focal adhesions and F-actin organization. MET1 cultures were left untreated (U), transfected with either non-targeting (NT) or with *FERMT2*-targeting siRNA. Sixty-six hours after transfection, the cells were trypsinized, re-seeded, cultured for 8 h and processed for immunofluorescence microscopy. Phospho-paxillin (P-Pax) immunoreactivity and Alexa Fluor 488®-conjugated phalloidin were used to visualize, respectively, focal adhesions and F-actin.

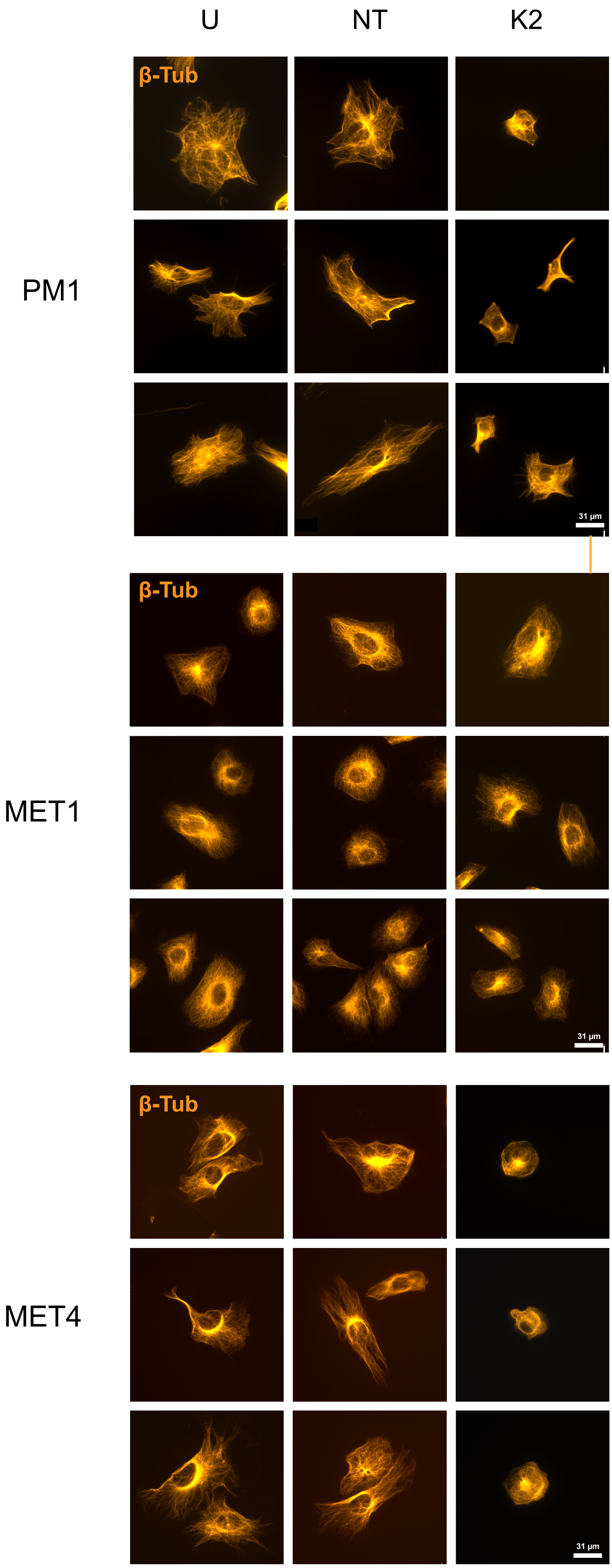

Supplemental Fig. S5. Effect of *FERMT2* silencing on microtubule organization. Cultures of the indicated cell lines were left untreated (U), transfected with either non-targeting (NT) or with *FERMT2*-targeting siRNA. Sixty-six hours after transfection, the cells were trypsinized, re-seeded, cultured for 8 h and processed for immunofluorescence microscopy using anti-β-tubulin antibodies.
